# Meetings of Societies

**Published:** 1913-12

**Authors:** 


### MEETINGS OF SOCIETIES.

### Bristol Medico-Chirurgical Society.

*Annual Meeting, October 8th,* 1913.

After a vote of thanks to the retiring President, Dr. Walter C. Swayne, had been passed, Dr. P. Watson-Williams took the chair and gave an introductory address (*see* page 289). Later a cordial vote of thanks was given to Dr. Watson-Williams for his able and interesting address.

The Honorary Secretary, Mr. A. J. Wright, read the following annual report :—

During the year twelve new members have been elected, and eight have resigned. There is now a total of 4 honorary and 171 ordinary members.

The balance from the previous year was £77 11s. 2d., and on this year there is a balance of £20 12s. 2d., making a total balance to date of £98 2s. 4d.

The average attendance at meetings has been 32.

The Society will again in the near future have to move to temporary premises during the reconstruction of the old Blind Asylum buildings.

The Hon. Librarian, Mr. C. K. Rudge, then read the following report :—

The Library of the Bristol Medico-Chirurgical Society now contains 12,254 volumes. During the year 302 volumes have been added, and 22 volumes sold. The Library is in regular receipt of 191 periodicals, 163 by exchange and 28 by purchase.

During the year 793 issues of books on loan were made to the members of the Society, and 53 volumes were obtained from Lewis's Library at the request of members.

The Bristol Medical Library, to which members of the Society have access, now contains 22,799 volumes, and 252 current periodicals, made up in the following manner :—

BOOKS.

| | |
|---|---|
| Bristol Medico-Chirurgical Society .. | 12,254 |
| University of Bristol .. .. .. | 8,782 |
| Bristol Royal Infirmary .. .. | 1,189 |
| Bristol General Hospital .. .. | 574 |
| Total .. .. | 22,799 |

CURRENT PERIODICALS.

| | | |
|---|---|---|
| Medico-Chirurgical Society | .. .. | 191 |
| University of Bristol | .. .. .. | 61 |
| Total | .. .. | 252 |

The following are the officers of the Society for the Session 1913–14: President—P. Watson-Williams, M.D. Lond; President-Elect—W. H. C. Newnham, M.B. Cantab. Committee (*ex-offlio*)—W. C. Swayne, M.D. Lond.; P. Watson-Williams, M.D. Lond. (Editor of *Journal*); C. K. Rudge, M.R.C.S. (Hon. Librarian); J. M. Fortescue-Brickdale, M.D. Oxon. (Editorial Secretary); A. J. Wright, M.B. Lond. (Hon. Secretary). (*Elected*)—R. Shingleton Smith, M.D. Lond.; G. Munro Smith, M.D. Bristol; G. Parker, M.D. Cantab.; I. Walker Hall, M.D. Vict.; F. Lace, F.R.C.S.; J. Lacy Firth, M.S. Lond.; J. O. Symes, M.D. Lond.; L. E. V. Every-Clayton, M.D. Lond.; Hon. Secretary, A. J. Wright, M.B. Lond.

**RECEIPT AND EXPENDITURE ACCOUNT OF THE BRISTOL MEDICO-CHIRURGICAL SOCIETY, SESSION 1912-13.**

| | £ | s. | d. |
|---|---|---|---|
| Balance from 1911–12 .. | 77 | 11 | 2 |
| Members' Subscriptions | 181 | 13 | 0 |
| Interest on Deposit .. | 2 | 10 | 2 |
| | £261 | 14 | 4 |

| | £ | s. | d. |
|---|---|---|---|
| University of Bristol, one Year's Rent .. .. | 52 | 10 | 0 |
| Honorarium (Farr) .. | 10 | 0 | 0 |
| Postage and typing .. | 24 | 9 | 0 |
| To *Journal* .. .. .. | 45 | 19 | 8 |
| Grant to Library .. | 30 | 0 | 0 |
| Telephone Rent .. .. | 5 | 16 | 10 |
| Lantern .. .. .. | 3 | 13 | 0 |
| Printing .. .. .. | 5 | 14 | 3 |
| Expenses of Meeting .. | 1 | 13 | 0 |
| University Marshal .. | 0 | 10 | 6 |
| Cheque Book .. .. | 0 | 2 | 6 |
| Petty Cash Disbursements .. .. .. | 3 | 3 | 4 |
| Balance in Bank, Sept. 30th, 1913 .. .. | 98 | 2 | 3 |
| | £261 | 14 | 4 |

We certify that we have examined the above account with the books, papers, and vouchers produced to us, and found the same correct.

CURTIS, JENKINS & Co.,
*Chartered Accountants.*

Bristol, *7th October*, 1913.

*November 12th*, 1913.

Dr. P. Watson-Williams, President, in the Chair.

The following new Rule was confirmed:—" The *Bristol Medico-Chirurgical Journal* shall have the exclusive right to claim for publication all papers read at Meetings of the Society if the Editorial Committee so desires"; and also such an alteration to Rule 6 as may allow of an afternoon meeting being held.

**A demonstration of apparatus for the production of intratracheal anæsthesia,** by Mr. F. E. Shipway (London), Mr. S. V. Stock, Mr. J. D. Fry and Mr. E. W. Hey Groves, was followed by a discussion (see pages 341 to 349).

Dr. Nixon read notes on a case of **Splenectomy for simple splenomegaly** (see page 325).

Mr. Groves read notes on a case of **Splenectomy in Banti's disease** (see page 331).—Dr. Waterhouse (Bath), asked if it was recommended that the enlarged spleen due to congenital syphilis should be removed. He had lately used salvarsan and neo-salvarsan in such a case, associated with pharyngeal ulceration. The ulceration had been cured, but the spleen was unaffected.—Dr. Fortescue-Brickdale discussed the use of the term Banti's Disease. He did not consider Mr. Groves's case to be one of ordinary Banti's Disease.—Dr. James Swain alluded to a case of splenectomy he had published in the *Bristol Medico-Chirurgical Journal,* in which the blood changes following the operation had been similar to those in Dr. Nixon's case.—Dr. Nixon, in replying, advocated splenectomy for all enlargements of the spleen causing trouble in the absence of myelogenous leukæmia.—Mr. Groves also advised removing the spleen in every case of enlargement where there were abdominal troubles present.

Mr. J. Lacy Firth gave the history of a case of **Abscess of the liver,** and showed the specimen. The patient, a man of 29, was first admitted to the Bristol General Hospital in February, 1913, with an abscess pointing in the epigastrium. He was operated upon an hour after admission, and a large quantity of pus evacuated from the left lobe of the liver. He left the hospital after a month, and returned to work. In October, having been doing ordinary work for five months or more, he was again admitted, complaining of constant cough, and pain in the back and left chest. He was intensely anæmic. A blood count showed secondary anæmia. By abdominal section a large abscess was opened and drained in the right lobe of the liver. The patient died a few days later. *Post-mortem* the healed scar of the first abscess was found, and also a third unopened abscess at the back of the liver, with its posterior wall close to the inferior vena cava. There was no peritonitis, but many ulcers of the amœbic dysenteric type were found in the large bowel. No history of dysentery could be obtained from the patient, and no symptoms of dysentery had been observed during his stay in the hospital. The case illustrated some of the difficulties met with at times in diagnosing abscess of the liver, for the liver had not been enlarged or tender, and the pain had been in the opposite side of the chest. The speaker advocated exploration by abdominal section in such cases, rather than the use of the exploring syringe.

J. Lacy Firth.
A. J. Wright, *Hon. Sec.*

---

### Local Medical Notes.

---

